# The impact of the COVID-19 pandemic on hospital admissions for psychiatric disorders: results from the multicentre study on the Italian population “COVID-19 and Mental Health” (CoMeH)

**DOI:** 10.1186/s12888-025-07076-9

**Published:** 2025-07-01

**Authors:** Massimiliano Aragona, Martina Ventura, Roberta Ciampichini, Anteo Di Napoli, Valeria Fano, Sara Leone, Martina Pacifici, Claudio Rosini, Caterina Silvestri, Fabio Voller, Alberto Zucchi, Christian Napoli, Alessio Petrelli

**Affiliations:** 1 National Institute for Health, Migration and Poverty (INMP), via di San Gallicano 25a, 00163 Rome, Italy; 2 Health Protection Agency (ATS), Bergamo, Italy; 3 Local Health Unit Roma 2, Rome, Italy; 4 IRCCS Regina Elena National Cancer Institute, Rome, Italy; 5 Tuscany Regional Health Agency (ARS), Florence, Italy

**Keywords:** Mental health, Inpatients, Psychiatry, Emigrants and immigrants, Hospitalization, Socioeconomic factors

## Abstract

**Aim:**

To evaluate the impact of the COVID-19 pandemic on hospital admissions for psychiatric disorders, with a focus on the patients’ socioeconomic and demographic characteristics and/or the diagnostic group and type of admission.

**Methods:**

Open cohort of individuals aged ≥ 10 years resident for at least two years in one of three large areas of Italy, covering about 6 million inhabitants (nearly 10% of the entire population). The outcome was the first admission for a psychiatric disorder (First Mental Health Admission: FMHA) during the study period (January 2018–December 2021). Municipality of residence, sex, census tract deprivation index, citizenship, type of admission and the diagnostic group were considered as covariates of interest. Incidence rate ratios (IRR) of FMHAs were estimated via an interrupted time series (ITS) analysis using a step-change negative binomial model. Moreover, an ITS analysis was conducted on the monthly number of FMHAs to evaluate the impact of COVID-19 on the temporal trend of FMHAs.

**Results:**

Of the 5,159,363 subjects enrolled, 11,171 had an FMHA in the study period. The incidence of FMHAs decreased after the outbreak of the pandemic, overall (from 0.169/100,000 to 0.147/100,000) and by sex, deprivation level and citizenship. Immigrants from high migration pressure countries (HMPCs) had a greater reduction in FMHAs during COVID-19 (IRR: 0.91, *p* < 0.01). A decrease in FMHAs was observed for all diagnoses, the only exceptions being for post-traumatic stress and related disorders, which increased. Involuntary admissions also increased abruptly at the outbreak of the pandemic (from pre-COVID IRR 0.98 to 1.32). Younger age (< 34) and high deprivation level were associated with higher risk of hospitalization. In the following post-outbreak period (March 2020-December 2021), a moderate but significant growing trend of FMHAs was observed, although not reaching the pre-pandemic levels. A more robust increase was found especially for patients with eating disorders, while FMHAs for patients with post-traumatic stress and related disorders decreased to the low pre-pandemic levels. Involuntary FMHAs also decreased in the post-outbreak period.

**Conclusions:**

The pandemic had a considerable impact on hospitalizations for psychiatric disorders. Hospitalization rates generally decreased, especially among immigrants. Younger people and those with high socioeconomic deprivation had a higher risk of hospitalization. PTSD diagnoses increased but only for a short period, while eating disorders tended to increase in the post-COVID period. In general, the effect of COVID on mental health hospitalizations appeared temporary. Longer follow-up surveys are needed.

**Clinical trial number:**

Not applicable.

**Supplementary Information:**

The online version contains supplementary material available at 10.1186/s12888-025-07076-9.

## Background

The COVID-19 pandemic considerably impacted the population worldwide on various levels, including a medical, social, and/ or economic impact. In terms of the impact of the pandemic on mental health, a significant increase in the number of suicides was expected, although the data on the correlation between suicidal behaviours and the pandemic outbreak are still controversial [1], and the relationship between these factors in the successive pandemic waves is complex [2]. Moreover, the impact on the mental health of the elderly population was also considerable [3]. Finally, the traumatic experience of the pandemic could have triggered the onset of post-traumatic stress disorder, with different post-traumatic stress symptoms depending on perceived risk and protection factors [4].

Some of the stress that impacted mental health was generated by news reports and the effects of social restrictions that the governments were often forced to adopt to reduce the spread of infection. In Italy, the first autochthonous case of COVID-19 was reported on 21 February 2020. A few days later, on 08 March, the Italian government imposed the first lockdown in Western countries: a nationwide restriction on the movement of the population, except for the purchase of food and other necessities (medicines, disinfectants, and so on), for essential workers and for health emergencies [5]. That summer, restrictions were eased, but in the winter of 2020 there was a surge in COVID-19 cases, with new restrictions applied. Over the subsequent two years, social measures were gradually eased, and normality returned.

Due to several factors, including social restrictions on movement, the COVID-19 pandemic also had repercussions on the accessibility of mental health services [6].

Regarding hospitalization for mental disorders, the evidence varies from nation to nation. In general, with some variability, the overall trend was a reduction in the number of new patients attending inpatient mental health facilities [7–10], with no differential impact on males or females [8, 10]. Regarding age, the evidence varies; some studies found a reduction only in older patients (≥ 65 years) [6], while others found no difference in terms of age [8]. The number of involuntary admissions either decreased or remained unchanged, depending on the study [7–10], and differences were also found concerning the diagnostic groups [8, 11]. Other differences in terms of the risk of psychiatric admission were reported for young migrants [12] and for individuals experiencing a worsening financial situation [13].

Overall, the available evidence suggests that the reduction in hospital admissions for mental disorders after the outbreak of the COVID-19 pandemic tended to persist over time. Finally, some differences between migrants and natives and in relation to socioeconomic factors have been reported, but to our knowledge, these important factors have not been examined in detail in population studies.

The aim of this study was to evaluate the impact of the COVID-19 pandemic on hospital admissions for psychiatric disorders. In particular, trends and rates of these admissions before and after the outbreak of the pandemic were analysed, with a focus on differences related to patients’ socioeconomic and demographic characteristics and/or to the diagnostic group and type of admission.

### Methods

This study is part of the larger *COVID-19 and Mental Health* (CoMeH) project [14] evaluating the impact of the COVID-19 pandemic on the use of mental health services in Italy, with a particular focus on socioeconomic and citizenship inequalities. The CoMeH project is a collaborative multicentre study promoted (designed and planned) by the National Epidemiologic Observatory for Equity in Health (OENES) of the National Institute for Health, Migration and Poverty (INMP) and conducted in three large Italian areas. The study was self-financed with INMP funds.

The Tuscany Regional Health Agency (RHA), the Bergamo Local Health Authority (LHA) and the Rome 2 LHA responded to a call to participate in the study, designed to include at least one region in northern Italy (the area most affected by the COVID-19 infection) and a sufficient geographical distribution. The geographical areas covered by the three participating centres represent almost six million beneficiaries (nearly 10% of the entire population) of the Italian National Health Service (NHS).

Within the CoMeH project, we retrospectively enrolled an open cohort of subjects aged ≥ 10 years resident for at least two years in one of the participating areas and assisted by an NHS general practitioner (GP). The enrolment period was planned from 01 January 2018 to 31 December 2024. Data of the present study are updated to 31 December 2021 [14].

The enrolled subjects exited the cohort at the end of the planned period (31 December 2021) or at time of death or at time of emigration, whichever occurred first. Given the open cohort design, individuals could re-enter the cohort through re-immigration to the resident population of the areas of the participating health centres.

### Outcome

We considered as outcome the first admission during the period of follow-up (January 2018 - December 2021) of patients at least 14 years old for a psychiatric disorder as first diagnosis leading to hospitalization (First Mental Health Admission: FMHA). Having included in the cohort subjects from age 10 years, and because the study covered four years (from 2018 to 2021), 14 years old was the minimum age needed to assess the outcome during the study period.

Data on hospitalizations were retrieved through a deterministic record linkage with the Italian hospital discharge database, which collects information on patients discharged from public and private hospitals, including diagnoses recorded using the International Classification of Disease, 9th Revision, Clinical Modification (ICD-9-CM) [15]. All hospitals located in the participating centres were considered. In Italy, mental health care is organized on a “territorial” basis. Each local health authority (LHA) has a mental health department, whose outpatient service (Mental Health Centre) decides when hospitalization is necessary. In Italy, many general hospitals include a psychiatric ward; admission to both public hospitals and private clinics must usually be authorised by the psychiatrist of the Mental Health Centre. An exception to this regards individuals accessing an emergency department (ED), where the hospital staff psychiatrist is called for a consultation and may decide on direct hospitalization. Private clinics usually do not have an ED and therefore do not see emergency psychiatric patients who may require hospitalization.

We included in this study all patients that had one of the following diagnostic codes of the ICD-9-CM as their first diagnosis: 291–292 and 303–305 (alcohol and substance disorders); 293–294 (transient psychic disorders due to other conditions); 295 (schizophrenia and related disorders); 296 and 311 (mood disorders); 297 (delusional disorders); 298–299 (other psychoses); 300 (anxiety and neurotic disorders); 301 (personality disorders); 307.1, 307.50 and 307.51 (eating disorders); 307.4 (sleep disorders); 307.8 (pain disorders); 308–309 (traumatic stress and adjustment disorders); 312.30 and 312.31 (impulse and gambling).

Only incident hospitalizations, identified as those events without any other admission for the same selection of psychiatric disorders in the two years prior to the event date, were considered as outcomes.

### Covariates

The sociodemographic information of the individuals enrolled in the cohort was obtained from municipal population registers and includes date of birth, date of death, date of emigration from or re-immigration to the residence area, municipality of residence, census tract, sex (male, female) and citizenship. Age at enrolment in the cohort was calculated using the date of birth and categorized into five groups (≤ 34, 35–64, 65–74, 75–84, 85+).

Socioeconomic status was determined using the national 2011 version of the census tract deprivation index, calculated as the sum of five standardized indicators and categorized in quintiles (Low, Middle-Low, Middle, Middle-High and High Deprivation). As census tracts are very small areas, the deprivation index can be considered a good proxy of individual socioeconomic status [16].

For the analysis of migration background, all the residents in Italy without Italian citizenship were considered as immigrants. As immigrants from highly developed countries (HDCs) accounted for only about 5% of the foreign resident population in the cohort, and they generally have a health profile comparable to that of the native population, they were included in the “Italians” group; immigrants from high migratory pressure countries (HMPCs) were included in the “immigrants” group [17].

The study period was divided into two phases: pre-COVID-19 (from January 2018 to February 2020) and post-COVID-19 (from the outbreak of the pandemic in March 2020 to December 2021). In this study the term “outbreak” of the pandemic is used to refer to the moment in which the first social restriction measures were adopted by the Italian government, at the beginning of March 2020.

The type of admission (planned, urgent or involuntary) and the group of diagnoses were used to characterize the hospitalizations. Regarding psychiatric diagnoses, the ICD-9-CM single codes were grouped into the following 15 general categories: Adjustment Disorders (all 309 except 309.81); Anxiety Disorders (293.84, 300.00-300.02, 300.09, 300.20-300.23, 300.29, 300.3, 300.5); Delirium/Mental Confusion (291.0, 292.81, 293.0, 293.1, 298.2); Dependencies (291–292, 303–305); Dissociation (300.10-300.15, 300.6); Eating Disorders (307.1, 307.5, 307.50, 307.51); Mood Disorders (293.83, 296, 298.0, 300.4, 309.1, 311); Personality Disorders (301); Post-Traumatic Stress and related Disorders (308, 309.81); unspecified Psychoses (293.81, 293.82, 298, 298.1, 298.4, 298.8, 298.9, 299.90, 299.91); Schizoaffective Disorder (295.7); Schizophrenia Spectrum (295, 295.0-295.6, 295.8-295.9, 297.0-297.9, 298.3); Sleep Disorders (307.4); Somatization (300.7, 300.8, 307.8); Others (293, 293.8, 293.9, 294, 294.8, 294.9, 300.16, 300.19, 300.9, 312.3).

### Ethics and privacy

The study protocol was approved by the Ethics Committee of the Italian National Institute of Health (Istituto Superiore di Sanità), protocol n. 0029105, 25 July 2022. The study was conducted in accordance with the Declaration of Helsinki [18].

Authorization for the use of anonymized data was obtained from the Data Protection Officer of all the participating centres per EU regulation 2016/679.

Privacy protection was ensured by assigning each individual a validated anonymous patient identifier so that multiple data sources could be linked, thereby providing the study with the complete care pathway of all the citizens resident in the three geographical areas involved in the study. Any personally identifiable information was hidden from individual records.

As this was a population-based study, individual consent to participate was deemed unnecessary according to national regulations; both the Ethics Committee of the Italian National Institute of Health and the data protection officers of all three centres waived the need for consent to participate.

### Statistical analysis

Baseline demographic and socioeconomic characteristics of the CoMeH study cohort and of the FMHAs identified during the follow-up are described as frequency and percentages.

To assess the effect of the pandemic on FMHAs, crude incidence rates per 100,000 person days, with 95%CI, were calculated overall and comparing the two time periods (pre-COVID-19 and post-COVID-19) by sex, deprivation level, citizenship and group of diagnoses.

In order to capture the impact of the pandemic on hospital admissions for psychiatric disorders, we conducted two types of analysis, both in the context of interrupted time series (ITS) [19].

In the first analysis, incidence rate ratios (IRR) of FMHAs with 95%CI were estimated via an ITS analysis using a step-change negative binomial model [20, 21]. The model was performed taking into account the presence of over-dispersion, with the log person time of follow-up for each group as offset. The model included age classes, sex, a binary period term (pre-pandemic and pandemic), citizenship and deprivation level. Additional terms comprised a linear time variable to account for long-term trends and a categorical calendar month variable to capture any seasonal effects. Moreover, to assess the effect of citizenship and deprivation level on FMHAs pre-COVID-19 and post-COVID-19, two interaction terms between citizenship and time period and between deprivation level and time period were included (Model 1). The first term provides a comparison of the difference in FMHA rates between immigrants from HMPCs and Italians + HDCs pre-pandemic to the difference in FMHA rates between immigrants from HMPCs and Italians + HDCs during the pandemic; the second provides a comparison between the difference in FMHA rates based on deprivation level pre-pandemic and the difference in FMHA rates based on deprivation level during the pandemic With the same purpose, the interaction between age and time period was also tested (Model 2).

In the second analysis, the impact of the pandemic on the temporal trend of FMHAs was investigated, estimating IRR, overall and stratified by sex, age groups, citizenship, deprivation level, type of admission and group of diagnoses, selecting only those diagnostic groups with at least 150 admissions. In this case, the ITS analysis was conducted on the monthly number of FMHAs. According to the procedure proposed by Schuengel et al. [22], data were detrended using Loess regression and smoothing [23], and seasonality was tested. If the presence of seasonal effects was identified, an adjustment was performed. The change in slope from the pre-pandemic to the pandemic period was tested using Poisson segmented regression with Newey-West standard errors to account for autocorrelation and heteroscedasticity [19]. The pre-COVID-19 trend, the immediate effect of COVID-19 on monthly trend of FMHAs (as a binary term) and the post-COVID-19 trend were evaluated.

All statistical analyses were performed using SAS 9.3 and R Studio (version 4.1.3).

## Results

Overall, 5,159,363 subjects were enrolled in the cohort between the 01 January 2018 and 31 December 2021. Table 1 shows the characteristics of the study population and of the FHMAs. During follow-up, 11,171 FMHAs were observed. Compared to the general population, the patients admitted to the hospital with a psychiatric diagnosis were younger (34.1% vs. 24.3% aged < 34 years), while the distributions by sex, deprivation level and citizenship were similar.

**Table 1:** Baseline characteristics of the study population and of first mental health hospital admissions (FMHAs)

Baseline characteristics	Population		First MH hospital admission	
	*N*	%	*N*	%
**Total**	5.159.363		11.171	0.22%
**Age-groups**				
≤ 34	1.254.290	24.3%	3.811	34.1%
35–64	2.510.617	48.7%	5.130	45.9%
65–74	664.706	12.9%	964	8.6%
75–84	506.405	9.8%	896	8.0%
85+	223.345	4.3%	370	3.3%
**Sex**				
Males	2.466.975	47.8%	5.359	48.0%
Females	2.692.388	52.2%	5.812	52.0%
**Deprivation level (quintiles)**				
Low	1.026.856	19.9%	2.277	20.4%
Middle-low	1.193.167	23.1%	2.628	23.5%
Middle	1.065.511	20.7%	2.289	20.5%
Middle- high	753.674	14.6%	1.685	15.1%
High	464.521	9.0%	1.114	10.0%
Missing data	655.634	12.7%	1.178	10.6%
**Citizenship**				
Italians	4.614.768	89.4%	10.093	90.4%
Immigrants from HDCs	22.650	0.4%	112	1.0%
Immigrants from HPMCs	446.334	8.7%	906	8.1%
Missing data	75.611	1.5%	60	0.5%
**Group of diagnoses**				
Delirium/Mental Confusion			728	6.5%
Dependencies			1.317	11.8%
Dissociation			100	0.9%
Eating Disorders			493	4.4%
Anxiety Disorders			364	3.3%
Sleep Disorders			12	0.1%
Mood Disorders			3.694	33.1%
Personality Disorders			804	7.2%
Schizoaffective Disorder			311	2.8%
Unspecified Psychoses			1.117	10.0%
Adjustment Disorders			733	6.6%
Post-Traumatic Stress and related Disorders			244	2.2%
Somatization			134	1.2%
Schizophrenic Spectrum			961	8.6%
Others			159	1.4%
**Type of admission**				
Scheduled			2.191	19.6%
Urgent			8.533	76.4%
Involuntary medical treatment			386	3.5%

Mood Disorders were the most frequent diagnoses (33.1%), followed by Dependencies (11.8%), unspecified Psychoses (10%), Schizophrenia Spectrum (8.6%) and Personality Disorders (7.2%); most of the hospitalizations were urgent (76.4%).

As shown in Table 2, crude rates of incidence of FMHAs decreased after the outbreak of the pandemic, overall and by sex, deprivation level and citizenship. Immigrants from HMPCs had higher rates compared to Italians in the pre-pandemic period and experienced a higher reduction in total hospitalization rates during the pandemic. Analysing rates by group of diagnoses, a decrease was observed for all diagnoses, the only exception being for post-traumatic stress disorders, which increased.

**Table 2:** FMHAs: crude incidence rates *100,000 person days by sociodemographic characteristics and group of diagnosis in pre- and post-COVID-19 periods

	Pre COVID-19			Post COVID-19		
	FMHAs		Crude incidence rate (95% CI)*100,000 person-days	FMHAs		Crude incidence rate (95% CI)*100,000 person-days
	*N*	%		*N*	%	
**Total**	**6**,**401**	**57.3**	0.169 (0.165-0.173)	**4**,**770**	**42.7**	0.147 (0.143-0.151)
**Sex**						
Males	3,091	48.3	0.171 (0.165-0.177)	2,268	47.5	0.147 (0.141-0.153)
Females	3,310	51.7	0.167 (0.161-0.173)	2,502	52.5	0.148 (0.142-0.154)
**Deprivation level (quintiles)**						
Low	1,281	20.0	0.169 (0.160-0.179)	996	20.9	0.154 (0.145-0.164)
Middle-low	1,533	23.9	0.174 (0.166-0.183)	1,095	23.0	0.146 (0.137-0.155)
Middle	1,291	20.2	0.165 (0.156-0.174)	998	20.9	0.149 (0.140-0.159)
Middle-high	974	15.2	0.176 (0.165-0.187)	711	14.9	0.151 (0.140-0.162)
High	637	10.0	0.186 (0.172-0.201)	477	10.0	0.165 (0.150-0.180)
**Citizenship**						
Italians+Immigrants from HDCs	5,816	90.9	0.169 (0.165-0.173)	4,337	90.9	0.148 (0.144-0.153)
Immigrants from HPMCs	510	8.0	0.175 (0.160-0.191)	396	8.3	0.146 (0.132-0.161)
**Group of diagnoses**						
Delirium/Mental Confusion	438	6.8	0.0135 (0.0123-0.0148)	290	6.1	0.0089 (0.0079-0.01)
Dependencies	817	12.8	0.0252 (0.0235-0.027)	500	10.5	0.0154 (0.0141-0.0168)
Dissociation	62	1.0	0.0019 (0.0015-0.0025)	38	0.8	0.0012 (0.0008-0.0016)
Eating Disorders	273	4.3	0.0084 (0.0075-0.0095)	220	4.6	0.0068 (0.0059-0.0077)
Anxiety Disorders	199	3.1	0.0061 (0.0053-0.0071)	165	3.5	0.0051 (0.0043-0.0059)
Sleep Disorders	9	0.1	0.0003 (0.0001-0.0005)	3	0.1	0.00009 (0.00002-0.00027)
Mood Disorders	2,081	32.5	0.0642 (0.0615-0.0671)	1,613	33.8	0.0498 (0.0474-0.0522)
Personality Disorders	448	7.0	0.0138 (0.0126-0.0152)	356	7.5	0.011 (0.0099-0.0122)
Schizoaffective Disorder	201	3.1	0.0062 (0.0054-0.0071)	110	2.3	0.0034 (0.0028-0.0041)
Unspecified Psychoses	582	9.1	0.018 (0.0165-0.0195)	535	11.2	0.0165 (0.0151-0.018)
Adjustment Disorders	427	6.7	0.0132 (0.012-0.0145)	306	6.4	0.0094 (0.0084-0.0106)
Post-Traumatic Stress and related Disorders	95	1.5	0.0029 (0.0024-0.0036)	149	3.1	0.0046 (0.0039-0.0054)
Somatization	93	1.5	0.0029 (0.0023-0.0035)	41	0.9	0.0013 (0.0009-0.0017)
Schizophrenic Spectrum	586	9.2	0.0181 (0.0167-0.0196)	375	7.9	0.0116 (0.0104-0.0128)
Others	90	1.4	0.0028 (0.0022-0.0034)	69	1.4	0.0021 (0.0017-0.0027)

* In some cases, percentages may not sum to 100% due to missing data

The results of the multivariate ITS analysis are shown in Table 3. Model 1 shows a reduction in accesses in the pandemic period compared to the pre-pandemic period (IRR = 0.80 *p* < 0.001), which was confirmed after adjusting for age, sex, deprivation level and citizenship. Instead, no significant interaction was detected between the COVID-19 period and deprivation level or between COVID-19 period and citizenship. All age classes showed lower probabilities of FMHAs compared to the youngest class (< 34). High deprivation level was associated with a higher risk of hospitalization, although at the limit of statistical significance.

**Table 3:** Results of ITS multivariate regression models. Adjusted incidence rate ratios and 95%CI of FMHAs

	Model1			Model2		
Predictors	IRR	CI	*p*	IRR	CI	*p*
**Time period (ref. Pre-COVID-19)**						
Post-COVID-19	**0.80**	**0.72–0.90**	**< 0.001**	**0.88**	**0.79–0.97**	**0.011**
Time (continuous)	**1.00**	**1.00–1.01**	**0.002**	**1**	**1.00–1.01**	**0.002**
**Study Month (ref. January)**						
February	1.01	0.92–1.12	0.831	1.01	0.92–1.12	0.831
March	0.98	0.89–1.09	0.760	0.98	0.89–1.09	0.762
April	0.91	0.82–1.01	0.067	0.91	0.82–1.01	0.066
May	1.04	0.94–1.14	0.467	1.04	0.94–1.14	0.467
June	1.05	0.95–1.16	0.361	1.05	0.95–1.15	0.363
July	1.07	0.97–1.18	0.159	1.07	0.97–1.18	0.159
August	0.97	0.88–1.07	0.539	0.97	0.88–1.07	0.529
September	1.03	0.94–1.14	0.506	1.03	0.94–1.14	0.515
October	0.97	0.88–1.07	0.515	0.97	0.88–1.07	0.507
November	0.98	0.88–1.08	0.649	0.98	0.88–1.08	0.638
December	**0.89**	**0.81–0.99**	**0.030**	**0.89**	**0.81–0.99**	**0.028**
**Deprivation Level (ref. Low)**						
Middle-low	1.02	0.94–1.10	0.663	0.99	0.93–1.04	0.624
Middle	0.96	0.88–1.04	0.295	0.96	0.91–1.02	0.216
Middle-high	1.02	0.93–1.11	0.718	1.00	0.93–1.06	0.936
High	1.08	0.98–1.19	0.112	**1.08**	**1.00–1.16**	**0.054**
**Citizenship (ref. Italians + HDCs)**						
Immigrants from HPMCs	0.93	0.84–1.02	0.117	**0.91**	**0.84–0.98**	**0.009**
**Sex (ref. Females)**						
Males	0.98	0.94–1.02	0.413	0.98	0.94–1.02	0.404
**Age groups (ref. ≤34)**						
35–64	**0.65**	**0.63–0.69**	**< 0.001**	**0.71**	**0.67–0.75**	**< 0.001**
65–74	**0.46**	**0.43–0.50**	**< 0.001**	**0.51**	**0.46–0.56**	**< 0.001**
75–84	**0.59**	**0.55–0.64**	**< 0.001**	**0.61**	**0.55–0.68**	**< 0.001**
85+	**0.69**	**0.61–0.77**	**< 0.001**	**0.81**	**0.71–0.93**	**0.003**
Deprivation Level*Time period			*p* = 0.6			
Middle-low	0.93	0.82–1.04	0.215			
Middle	1.01	0.90–1.14	0.856			
Middle-high	0.96	0.84–1.09	0.517			
High	0.98	0.85–1.14	0.831			
Citizenship *Time period			*p* = 0.6			
Immigrants from HPMCs	0.96	0.83–1.11	0.557			
Age groups*Time period						*p* < 0.001
35–64				**0.84**	**0.77–0.92**	**< 0.001**
65–74				**0.81**	**0.70–0.95**	**0.009**
75–84				0.92	0.79–1.08	0.309
85+				**0.61**	**0.47–0.79**	**< 0.001**

The results of the interaction between age and COVID-19 showed a significant effect modification by age (Model 2): for all age categories, the effect of COVID-19 on FMHAs was stronger than that observed in the youngest age class (IRR = 0.88, 95%CI 0.79–0.97) (≤ 34). In particular, the effect of COVID-19 on FMHAs for those aged 35–64 was 14% higher (IRR = 0.74, 95%CI 0.67–0.81), 17% for those aged 65–74 (IRR = 0.71, 95%CI 0.61–0.83), 7% for 75–84 (IRR = 0.81, 95%CI 0.69–0.95) and 34% for 85+ (IRR = 0.54, 95%CI 0.42–0.69).

**Fig. 1:**
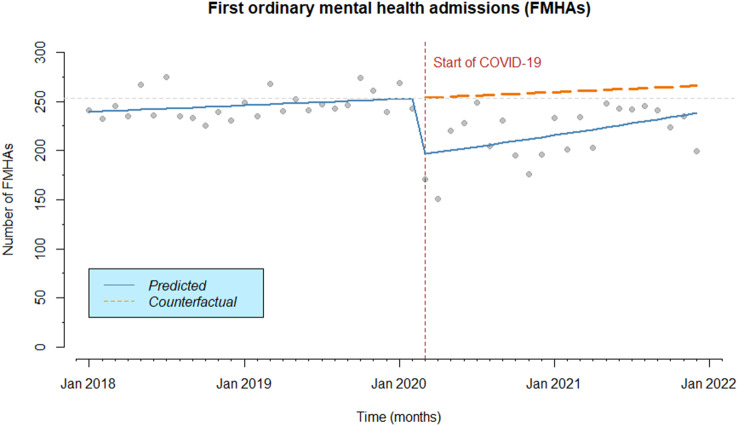
Number of monthly FMHAs before and during the COVID-19 pandemic. Vertical dashed line: introduction of restrictions. Continuous line: trend over the years. Dashed line: counterfactual scenario. Horizontal dashed line: pre-pandemic level

Table 4 shows the results of the ITS analysis conducted on the trend of the number of monthly FMHAs, overall and stratified by sex, citizenship, deprivation level, diagnostic group and type of admission. Overall, the average number of FMHAs was 232.7 per month (SD = 25.8), with a significant drop of 23% (IRR = 0.77, 95%CI 0.67–0.89) in all FMHAs, coinciding with the outbreak of the COVID-19 pandemic (March 2020). In the subsequent period, up to 31 December 2021, a moderate but significant growing trend (IRR = 1.01, 95% CI 1.002–1.017) was observed, although it did not reach the pre-pandemic levels (Table 4; Fig. 1).

**Table 4:** Results of ITS analysis of the monthly number of admissions, by demographic and socioeconomic variables, type of admission and group of diagnosis

FMHA characteristics	Mean (per month)	SD	Trend pre COVID-19	COVID-19 immediate effect at the outbreak	Trend post COVID-19
			IRR (95%CI)	IRR (95%CI)	IRR (95%CI)
**Total**	232.7	25.8	1.002 (1.000-1.004)	0.770 (0.667–0.889)	1.010 (1.002–1.017)
**Sex**					
Males	111.7	14.7	1.002 (0.999–1.005)	0.785 (0.669–0.920)	1.007 (0.996–1.018)
Females	121.1	16.1	1.003 (1.000-1.005)	0.757 (0.703–0.814)	1.011 (1.006–1.016)
**Age classes**					
14–34	79.4	12.4	1.012 (1.008–1.016)	0.738 (0.604–0.903)	1.017 (1.014–1.019)
35–64	106.9	15.0	0.999 (0.996–1.002)	0.870 (0.733–1.031)	0.998 (0.986–1.010)
65–74	20.1	4.5	0.998 (0.995–1.002)	0.680 (0.602–0.767)	1.017 (1.007–1.028)
75–84	18.7	5.4	1.003 (0.993–1.013)	0.541 (0.415–0.706)	1.030 (1.013–1.047)
85+	7.9	3.8	0.974 (0.961–0.987)	0.581 (0.395–0.854)	1.013 (0.991–1.036)
**Deprivation level (quintiles)**					
Low	47.4	7.4	1.004 (1.001–1.008)	0.759 (0.680–0.848)	1.012 (1.005–1.019)
Middle-low	54.8	8.6	0.997 (0.991–1.003)	0.817 (0.682–0.978)	1.006 (0.996–1.016)
Middle	47.7	7.7	1.005 (1.000-1.010)	0.793 (0.644–0.977)	1.007 (0.994–1.020)
Middle- high	35.1	8.1	1.003 (1.002–1.005)	0.694 (0.670–0.719)	1.015 (1.013–1.017)
High	23.2	5.1	1.000 (0.996–1.004)	0.783 (0.663–0.925)	1.010 (0.998–1.022)
**Citizenship**					
Italians + HDCs	211.5	23.7	1.002 (1.000-1.004)	0.767 (0.666–0.884)	1.010 (1.000-1.020)
Immigrants from HPMCs	18.9	4.7	1.006 (1.001–1.011)	0.805 (0.706–0.918)	1.005 (0.998–1.013)
**Group of diagnoses**					
Delirium/Mental Confusion	15.2	4.7	0.991 (0.981-1.000)	0.582 (0.447–0.759)	1.035 (1.020–1.049)
Dependencies	27.4	7.1	1.003 (0.997–1.009)	0.625 (0.462–0.843)	1.010 (0.993–1.027)
Eating Disorders	10.3	3.1	1.004 (1.002–1.006)	0.651 (0.619–0.684)	1.028 (1.025–1.031)
Anxiety Disorders	7.6	2.6	0.991 (0.985–0.998)	0.729 (0.649–0.819)	1.034 (1.029–1.039)
Mood Disorders	77.0	11.4	1.005 (1.002–1.009)	0.727 (0.644–0.822)	1.014 (1.004–1.024)
Personality Disorders	16.8	3.9	1.003 (0.998–1.008)	1.018 (0.940–1.102)	0.989 (0.986–0.993)
Schizoaffective Disorder	6.5	3.5	0.998 (0.974–1.003)	0.825 (0.569–1.194)	0.992 (0.964–1.021)
Unspecified Psychoses	23.3	5.3	1.001 (0.997–1.005)	0.970 (0.873–1.079)	1.009 (1.004–1.014)
Adjustment Disorders	15.3	4.1	1.011 (1.006–1.016)	0.647 (0.518–0.808)	1.012 (0.999–1.024)
Post-Traumatic Stress and related Disorders	5.1	3.1	1.021 (1.009–1.032)	1.973 (1.188–3.276)	0.972 (0.942–1.004)
Schizophrenic Spectrum	20.0	5.1	0.993 (0.982–1.004)	0.972 (0.776–1.216)	0.986 (0.973–0.998)
Others	3.3	1.6	1.025 (1.016–1.034)	0.621 (0.466–0.829)	1.007 (0.986–1.029)
**Type of admission**					
Scheduled	45.7	10.9	1.007 (1.004–1.009)	0.558 (0.501–0.622)	1.018 (1.010–1.026)
Urgent	177.8	17.6	1.003 (1.000-1.005)	0.816 (0.714–0.932)	1.009 (1.000-1.017)
Involuntary medical treatment	8.0	3.4	0.985 (0.972–0.999)	1.323 (0.853–2.053)	0.972 (0.937–1.008)

**Fig. 2:**
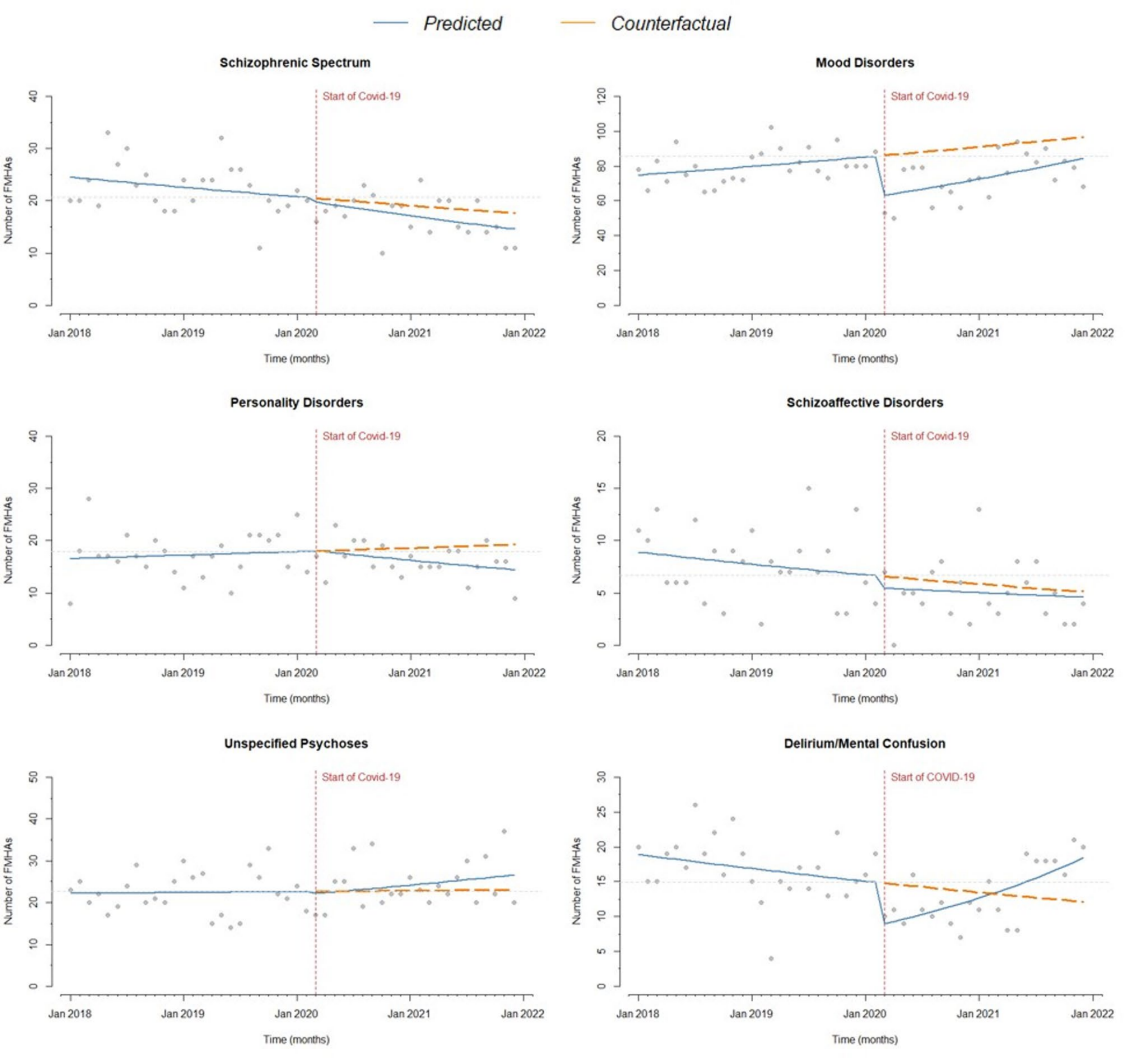
Number of monthly FMHAs before and during the COVID-19 pandemic. Diagnostic groups (I). Vertical dashed line: introduction of restrictions. Continuous line: trend over the years. Dashed line: counterfactual scenario. Horizontal dashed line: pre-pandemic level

**Fig. 3:**
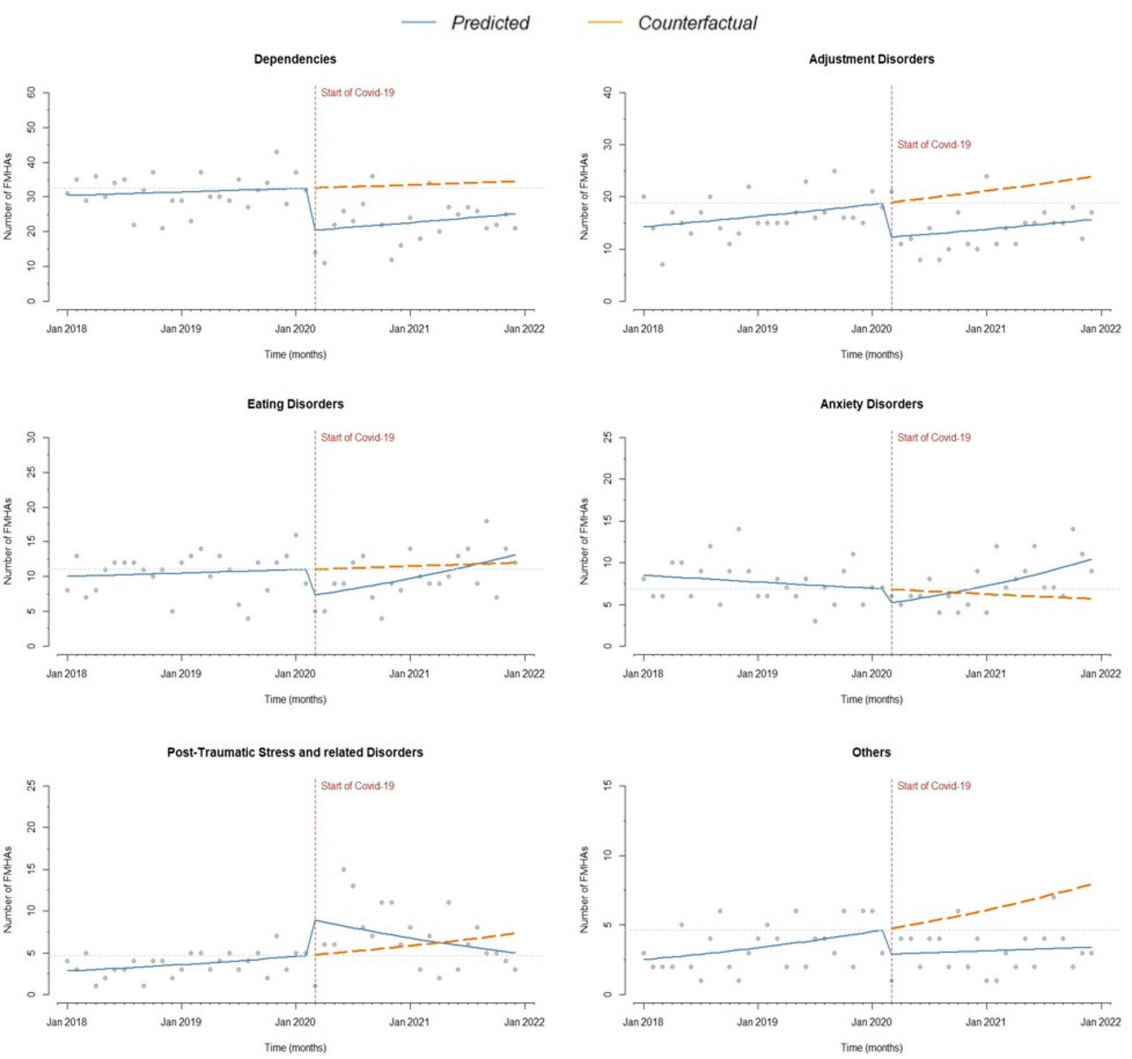
Number of monthly FMHAs before and during the COVID-19 pandemic. Diagnostic groups (II). Vertical dashed line: introduction of restrictions. Continuous line: trend over the years. Dashed line: counterfactual scenario. Horizontal dashed line: pre-pandemic level

Relevant differences were found by diagnostic group (Table 4; Figs. 2 and 3). A significant drop in FMHAs at the outbreak of the pandemic was observed for delirium/mental confusion (-42%), dependencies (-38%), adjustment disorders (-35%), eating disorders (-35%), anxiety and mood disorders (both − 27%). In patients with schizophrenia, schizoaffective disorder, unspecified psychoses and personality disorders, changes were not significant. In countertendency, post-traumatic stress and related disorders showed a strong increase in FMHAs (+ 97%).

The dynamics of FMHAs in the subsequent period (up to 31 December 2021) also showed considerable differences: a significant monthly increase in FMHAs was observed for unspecified psychoses, eating disorders, anxiety disorders, mood disorders and delirium/mental confusion.

Schizoaffective disorder showed a flat post-outbreak trend but in line with a decreasing trend that had already started before the pandemic outbreak. Personality disorders and schizophrenia showed a decreasing trend post-outbreak; in December 2021, the number of FMHAs was below both the pre-pandemic level and the counterfactual line. In the case of schizophrenia, the decreasing trend had already started before the pandemic, while the pre-pandemic trend for personality disorders had been stable. Finally, post-traumatic stress and related disorders showed a peculiar trend, with a reduction in this period, returning to pre-pandemic levels largely below the counterfactual line.

As shown in Table 4 and Supplementary Figs. 1–4, when analysing differences by sex, citizenship and deprivation level, the trends were similar to those observed for the general population, in both the COVID-19 immediate effect and the subsequent trends. The analysis by age group showed a stronger COVID-19 immediate effect in people aged ≥ 75.

**Fig. 4:**
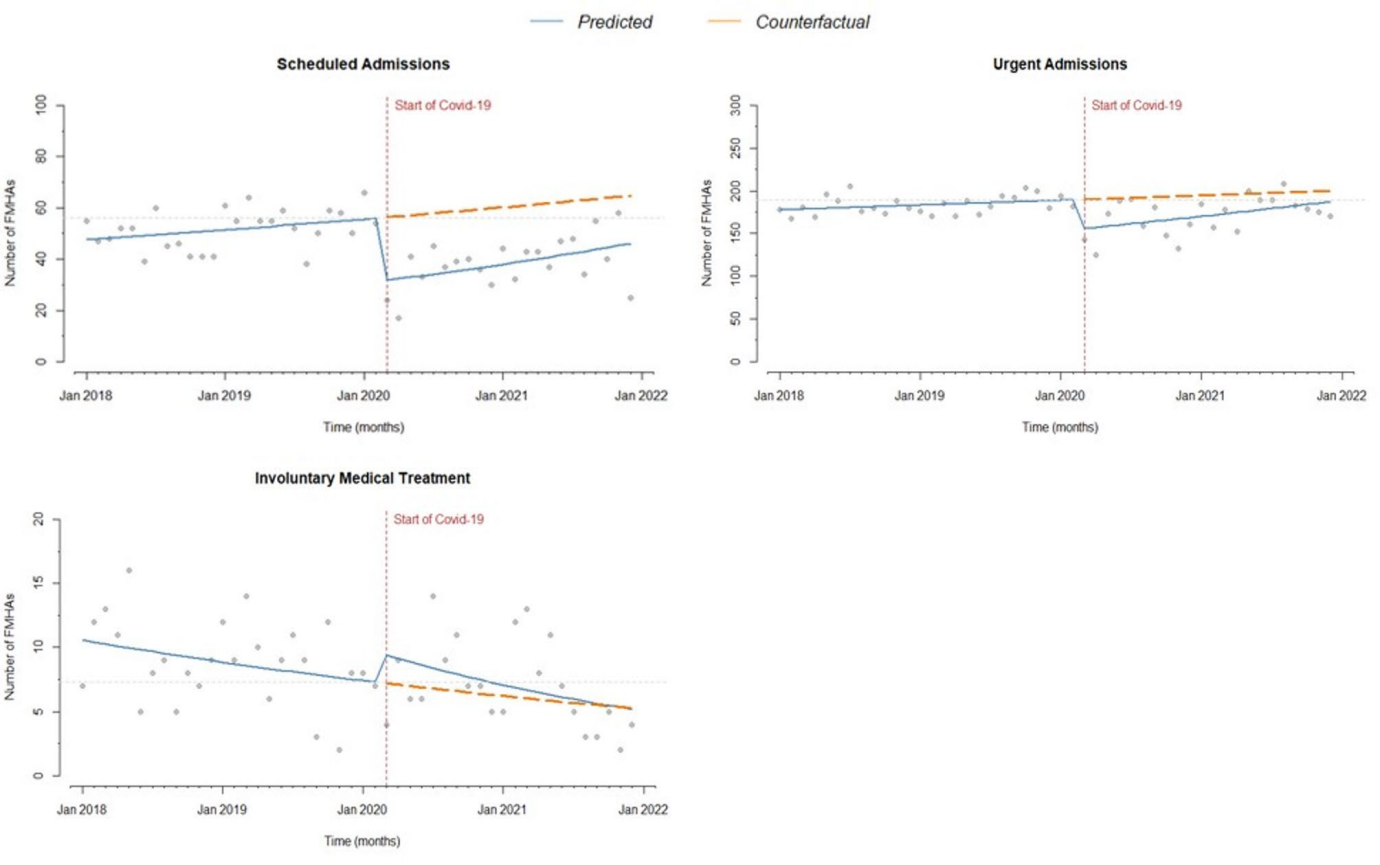
Number of monthly FMHAs before and during the COVID-19 pandemic. Type of admissions. Vertical dashed line: introduction of restrictions. Continuous line: trend over the years. Dashed line: counterfactual scenario. Horizontal dashed line: pre-pandemic level

As shown in Table 4; Fig. 4, there was a decrease in both planned and urgent FMHAs at the outbreak of the pandemic (-44% and − 18%, respectively), while there was a significant increase (+ 32%) in involuntary FMHAs. In the post-outbreak pandemic period up to 31 December 2021, there was a significant increasing trend only for planned admissions, although they were still below the pre-pandemic level. In the same period, urgent admissions returned to pre-pandemic levels but below the counterfactual level. Finally, involuntary FMHAs decreased in the post-outbreak period, in line with the counterfactual trend.

## Discussion

Our study shows that the outbreak of the COVID-19 pandemic was associated with an overall reduction in first admissions in hospitals for mental disorders. Many factors may have played a role in determining this result, despite the fact that an increase in hospitalizations due to the unavailability of alternative treatments in outpatient services was to be expected. Among these possible factors were a reduction in the number of beds in the psychiatric ward in some hospitals, difficulty in reaching hospitals, whose access points were busy with COVID-19 patients, the blocking of waiting lists for new scheduled admissions, fear of going to hospitals due to the increased risk of being infected, and so on [7–9, 24]. This overall reduction was confirmed even after adjusting for age, sex, deprivation level and citizenship. In line with previous studies [8, 11], the reduction in FMHAs was not significantly different between males and females.

The younger age of hospitalized patients in our study may be due to the exclusion of dementia from the diagnoses considered, as dementia is mainly treated by neurologists or geriatricians in Italy, with a negligible impact on psychiatric hospitalizations. Younger patients (age < 34) had a significantly lower reduction in FMHAs compared to older people, albeit within an overall reduction. This finding is in line with Hamlin et al. [11] but contrasts with Kim et al. [8], who found no differences related to the age of patients.

During the observation period, high deprivation level was associated with a higher risk of hospitalization, consistently with the international literature, which shows worse mental health among people living in more deprived areas [25]. Further studies using indicators of multiple dimensions of social status and their relationship to mental health services accessibility are needed to investigate how socioeconomic conditions changed during the pandemic.

Our results show that migrants from HMPCs had lower rates of FMHAs than did Italians and immigrants from HDCs, both before and after the outbreak of COVID-19. Immigration in Italy is a relatively recent phenomenon, so immigrants tend to be younger than Italians and thus have better health conditions, which could explain their lower use of mental health services [26]. Moreover, the reduction in psychiatric hospitalizations of immigrants from HMPCs in our study was greater during the outbreak of the pandemic, but the slope of the post-outbreak pandemic increase curve was also less steep compared to that for Italians and immigrants from HDCs. This finding is in line with previous pre-pandemic research showing that immigrants from HMPCs usually have lower rates of FMHAs compared to the Italian population [27]. This result has been interpreted as related to the “healthy immigrant effect” [28], although the authors stressed that the dynamics underlying psychiatric hospitalization was rather complex (depending on the prevalence of mental disorders in this population and on different accessibility to services and care pathways) and also that admission rates are not uniform in the population of foreign origin [27].

During the pandemic, another study conducted in an Italian setting showed an increase in psychiatric admissions among migrants, especially those in the youngest age group, who were also at higher risk of involuntary treatment [12]. Our findings apparently do not confirm this evidence, but in light of the methodological differences (e.g., Tarricone et al.’s study [12] covers a different Italian area and a shorter period of time), future in-depth studies on immigrants are needed.

Regarding the types of hospital admission, before the outbreak of the pandemic, planned and urgent admissions had a similar slightly increasing trend, while involuntary admissions were decreasing. With the onset of the pandemic, planned and urgent admissions dropped abruptly, while involuntary admissions increased. Finally, the post-outbreak pandemic trend returned to the pre-pandemic dynamic. Our evidence of a temporary abrupt increase in involuntary treatments during the outbreak of the pandemic is in line with one German study [29], while it contrasts with previous reports of unchanged or reduced involuntary admission in the COVID period [10–12, 26]. Our findings are in line with the expectation that during the COVID emergency, planned or urgent hospitalizations would be more difficult to organize, while involuntary treatment in cases of a psychiatric emergency would not encounter the same barriers to access. A study by Di Lorenzo et al. [30] indirectly confirms this expectation; although they did not report an increase in involuntary admissions, they still found a relevant reduction during the pandemic in the number of voluntary admissions, so that involuntary admissions in 2022 became prevalent.

Finally, the impact of the pandemic restriction measures appeared to vary by diagnostic group. Overall, there was a general drop in FMHAs at the outbreak of the pandemic for all diagnoses, with the exception of post-traumatic stress and related disorders, which sharply increased at the outbreak, then dropped in the subsequent period up to 31 December 2021, the robust increase in the initial period being only temporary. Regarding the other diagnostic groups, there was a general return towards the pre-pandemic levels in the post-outbreak period, with a more or less rapid increase depending on the diagnostic group. Anxiety disorders, eating disorders and delirium/mental confusion showed the most rapid increases.

These findings differ from previous studies, which found substantially stable patterns of weekly admissions around the COVID-19 outbreak [31], slight, non-significant reductions in hospital admissions for mood disorders and stress-related disorders [11], or an increasing pattern for bipolar disorder, depression and anxiety disorders [8]. The non-significant differences in FMHAs in the case of psychoses are in line with Hamlin et al. [11], who also found a significant decrease in admissions for substance use disorders, as confirmed in our study. However, their report of a significant increase in personality disorders [11] was not confirmed by our data.

Eating disorders showed a rapid post-outbreak increase in FMHAs that, at 31 December 2021, were above both the pre-pandemic incidence and the counterfactual line. This finding is in line with the evidence that patients with eating disorders experienced worsening symptomatology and increased isolation, with an upsurge in urgent care [32] and hospital admissions as a result of the timing of COVID-19 pandemic [33]. Further studies should identify vulnerable groups among the different kinds of eating disorders and study the long-term consequences of the pandemic period [34].

Regarding psychoses, schizophrenia showed a post-outbreak reduction in the trend of FMHAs, in line with Kim et al. [8], while unspecified psychoses increased significantly, with an incidence of FMHAs in December 2021 that was above the pre-pandemic incidence. Although it is not certain that the two diagnostic concepts are equivalent, it is interesting that another study found an increase in patients with “acute polymorphic psychotic disorders” [29]. The reasons for the observed opposite trend (reduction in schizophrenia and increase in unspecified or polymorphic psychoses) are not clear, and further research is needed to understand whether this was only an artefact due to the way the diagnoses were made or whether it is a signal that something is changing in the way patients are presenting their psychotic symptoms.

It was expected that the pandemic would have a significant impact on patients with personality disorders; in these patients, social distancing and loneliness could have caused anxiety of being abandoned, social withdrawal and feelings of emptiness [35]. However, this effect was not evident in the rates of FMHAs in our population, with incidence in December 2021 remaining below both the pre-pandemic levels and the counterfactual line. Further research using other proxies to study this possible effect is needed.

As expected, the COVID-19 emergency was responsible of a collective anxiety that was better managed once the authorities disseminated messages to reduce the level of uncertainty around the evolving COVID-19 situation [36]. In general, it was estimated that cases of anxiety disorders increased by 25% globally due to the pandemic [37]. However, thanks to the induction of resilience capabilities, the acute increase in anxiety at the pandemic outbreak could have declined over time, returning to pre-pandemic levels [38], suggesting that these findings may be overestimated [39]. Hospitalizations are not the best proxy to study anxiety because it usually does not require inpatient treatment. However, some effect was detected even at this level, considering that we found that the pre-pandemic trend of a reduction in FMHAs for anxiety disorders was inverted, with a significant rapid increase after the outbreak of the pandemic. As, to our knowledge, there are no studies specifically addressing possible severe forms of pandemic-induced anxiety, further longitudinal research on this is needed.

Symptoms of post-traumatic stress were largely expected due to the relevant impact of fear of COVID worldwide, and PTSD was in fact reported to have increased both in prospective and retrospective symptom analyses [40]. Despite the low number of FMHAs for post-traumatic stress and related disorders in our population, their significant increase (+ 97%) at the beginning of the pandemic, followed by a post-outbreak return to the pre-pandemic levels, is interesting because it confirms an acute post-traumatic impact of the pandemic that was only temporary, at least for the most severe forms, which required hospitalization. This finding is in line with Bourmistrova et al.’s [38] long-term reduction in the effect of the pandemic on PTSD in the population. Because there are relevant differences in rates of PTSD in specific subgroups, including children, adolescents, COVID-19 survivors and health professionals [41], further, more in-depth studies addressing the characteristics of the patients hospitalized with a diagnosis of PTSD and related disorders are needed.

Finally, we observed a rapid increase in FMHAs for delirium/mental confusion in the post-pandemic period. This finding is partly similar to the increase in cases of “delirium superimposed on dementia”, reported in another study [29]. The reasons behind this finding are not clear, and the group is too heterogeneous to permit explanatory hypotheses.

### Strengths and limitations of the study

To our knowledge, this is the first longitudinal study investigating the impact of the pandemic on hospital admissions for psychiatric disorders in Italy. Our study is based on a large population-based cohort followed up longitudinally through a powerful approach that allowed us to evaluate the large amount of information regarding sociodemographic and clinical conditions of the population cohort and their hospital admissions.

Using the Italian hospital information system allowed us to analyse the diagnostic groups that led to a hospitalization. To strengthen the specificity of the study, we decided to include in the analysis only mental disorders registered as “first diagnosis” in the ICD-9-CM hierarchical coding system. This choice made it possible to accurately identify the outcome of interest and to exclude those patients with psychiatric disorders who were hospitalized for other causes. The possible consequent limitation is that patients with mental disorders registered in the following hierarchical positions are not considered.

A limitation of our study is the relatively short follow-up of the cohort, as only the data up to the end of 2021 were available, which did not allow us to investigate the long-term effect of the pandemic. However, we were able to cover the most difficult phases of the pandemic. Moreover, as the CoMeH project plans to extend the follow-up until 2024, we will soon be able to study the long-term effect of the pandemic on hospital admissions for psychiatric disorders in Italy.

A second possible limitation concerns the generalizability of our results to other countries. In Italy, mental health care is based on the territorial model described in the Methods above; outpatient services and other territorial facilities directly treat patients, thereby reducing the need for hospitalization. Accordingly, the Italian National Health System has the lowest number of psychiatric care beds per 100,000 population in Europe. The reader should consider the wide inter-country variability of hospitalization rates for psychiatric disorders.

The generalizability of our results to other countries is also limited by the fact that Italy was one of the nations most severely hit by the pandemic worldwide and also one with the most severe government-imposed restriction measures.

Another limitation is that we used an area-based indicator, i.e., the census tract deprivation index, as a proxy to study individuals’ socioeconomic status, which may have introduced ecological bias. Consequently, we have no direct information on the subjects’ *individual* socioeconomic status, such as income or education level. Such individual information is important because it has been shown that many individual social factors, such as living alone and low or decreased income during the pandemic, were associated with exacerbating barriers to accessing mental health care [42].

The use of citizenship as a proxy for immigrant status could result in a residual information bias. In fact, according to Italian legislation, individuals born in Italy to non-Italian citizens are considered foreigners until the age of 18 years, while individuals born abroad can obtain Italian citizenship if they are descendants of Italian ancestors. These two facts slightly influenced the selection of the immigrant population: while boys and girls born in Italy were included as immigrants, people born abroad were included in the Italian population. Moreover, enrolling only subjects who were resident in the catchment areas of the study could not include undocumented migrants and migrants without a formal residence.

Furthermore, it should be mentioned that the case definition for incident cases (i.e., absence of hospitalization for psychiatric disorders in the previous two years) probably led us to overestimate the incidence in that this is a relatively brief period for excluding previous hospitalizations.

Another limitation of the study is that a correlation with the stringency index [43], which is a composite measure based on indicators including school and workplace closures and travel bans, was not performed.

Finally, although our population cohort covers about 6 million beneficiaries (nearly 10% of the entire Italian population), it is made up of residents in northern and central Italy. It is possible that the lack of coverage of any area located in the south of Italy could have introduced a bias in overestimating the impact of COVID-19, which more strongly affected the regions in the North.

### Practical implications

Overall, the main practical implications of this study are the followings:

1. Considering that we observed a general reduction in first hospital admissions for mental disorders, implementing alternative treatments to hospitalization for mental disorders will be important in the event of similar public health emergencies in the future. For example, maintaining access to outpatient services, in person and/or online, is crucial to reducing possible drug withdrawal symptoms, which can increase the risk of hospitalization. The evidenced increase in involuntary admissions shows that psychiatric emergencies were not sufficiently prevented in the pandemic period, stressing the importance of this point in future pandemic plans.
2. As migrants from poor countries, younger and older individuals, and those with socioeconomic deprivation were the groups more affected by COVID-19, they should be the target of proactive offers of psychological and psychiatric support aimed at reducing distress and the barriers to mental health services access in future crises.
3. The increase in hospitalizations for PTSD at the outbreak of the pandemic suggests that specific outpatient services for the treatment of traumatic experiences should be organized now so as to be ready to intervene in the event of new crises. Online interventions would be easy to implement in this context and would contribute to reducing the traumatic effect of the pandemic experience, preventing the need of hospitalization.
4. Finally, our study shows that eating disorders are those with the most relevant rebound of hospitalizations in the post-pandemic period, suggesting that treatment programmes focusing on its impact on the eating habits of the population should be organized. Moreover, studies specifically addressing this point should consider what preventive strategies could be organized in case of future crises.

## Conclusions

This study is part of the CoMeH project, designed to investigate the impact of the pandemic on mental health more comprehensively by evaluating all the main mental health services, including hospitalization, emergency care, outpatient care, psychiatric residential facilities and psychopharmacological prescriptions.

Regarding hospitalization for mental disorders during the pandemic, we found a general drop in hospitalizations at the outbreak of the COVID-19 emergency, followed by an increase in the period from the end of March 2020 to 31 December 2021 that, in many instances, remained below the expected trend. In particular, the drop in hospitalizations in the initial period of the pandemic was higher in older patients, migrants from high migratory pressure countries and patients with planned/urgent admissions. High deprivation level was associated with a higher risk of hospitalization. Finally, in countertendency, hospitalizations of patients with involuntary admissions and of patients with post-traumatic stress and related disorders increased at the outbreak of the pandemic.

In the subsequent period, hospitalizations generally tended to increase, more slowly in immigrants and more rapidly in patients with eating disorders, while hospitalizations of patients with involuntary admissions and patients with post-traumatic stress and related disorders decreased to pre-pandemic levels.

Overall, this study suggests that the pandemic had a considerable impact on hospitalizations for mental health diagnoses. However, the effect appeared temporary even in those patients who seemed most affected (e.g., those with post-traumatic stress and related disorders), suggesting that the expectations of a massive increase in mental health problems due to the pandemic may have been overemphasized, at least in the short term. Because it is possible that long-lasting effects will be more evident in longer follow-up surveys, further studies are needed to track these trends in the next few years.

## Electronic supplementary material

Below is the link to the electronic supplementary material.

Supplementary Material 1

Supplementary Material 2

Supplementary Material 3

Supplementary Material 4

## Data Availability

Data that support the findings of this study cannot be shared openly but are available on request writing to the correspondance author of this manuscript.

## Abbreviations

CI: Confidence Interval
CoMeH: Name of the study “COVID-19 and mental health”
COVID-19: Coronavirus disease 2019
ED: Emergency department
EU: European union
FMHA: First mental health admission
GP: General practitioner
HDCs: Highly developed countries
HMPCs: High migration pressure countries
ICD-9-CM: International classification of disease, 9th revision, clinical modification
INMP: Italian national institute for health, migration and poverty
IRR: Incidence rate ratios
ITS: Interrupted time series analysis
LHA: Local health authority
NHS: Italian national health service
OENES: National epidemiologic observatory for equity in health
PTSD: Post-traumatic stress disorder
RHA: Regional health agency
SD: Standard deviation
