# Supplementary material for: The impact of the COVID-19 pandemic on hospital admissions for psychiatric disorders: results from the multicentre study on the Italian population “COVID-19 and Mental Health” (CoMeH)
Source: BMC Psychiatry. 2025 Jul 1;25:633. doi: 10.1186/s12888-025-07076-9 (PMC12220136; doi:10.1186/s12888-025-07076-9)
Supplement: Supplementary file 1 — Supplementary Material 1 [file 12888_2025_7076_MOESM1_ESM.docx]

**Supplementary Figure 1. Number of monthly FMHAs before and during the COVID-19 pandemic. Sex.**


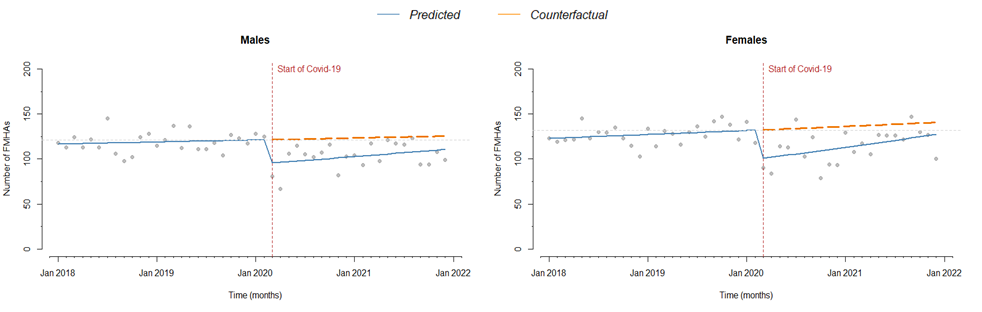


Vertical dashed line: introduction of restrictions. Continuous line: trend over the years. Dashed line: counterfactual scenario. Horizontal dashed line: pre-pandemic level.
